# Assessment of cannabis-related health impacts in Morocco: A decade-long epidemiological study

**DOI:** 10.1192/j.eurpsy.2025.1483

**Published:** 2025-08-26

**Authors:** O. Erefai, A. Soulaymani, S. Elkafssaoui, S. Hmimou, S. Irnat, S. Saidou, H. Chaoui, R. Soulaymani-Bencheikh, H. Hami

**Affiliations:** 1 Higher Institute of Nursing Professions and Health Techniques; 2 Royal School of Military Health Service, Rabat; 3 Laboratory of Biology and Health, Faculty of Science, Ibn Tofail University, Kenitra; 4 Moroccan Poison Control Center, Rabat, Morocco

## Abstract

**Introduction:**

The rise in cannabis consumption in Morocco, paralleling global patterns, has heightened concerns over its potential repercussions on mental and physical health, as well as its broader societal implications.

**Objectives:**

This study aims to delineate the demographic and clinical characteristics of individuals affected by cannabis toxicity and elucidate the details of their exposure scenarios in Morocco.

**Methods:**

We conducted a retrospective analysis of cannabis poisoning incidents from 2008 to 2017, encompassing both occasional and habitual users. Data were sourced from the Moroccan Poison Control Center (MPCC), providing a comprehensive national perspective.

**Results:**

During the study period, 553 cases of cannabis poisoning were reported, with a gender distribution of 19.3% female and 80.7% male. Notably, 245 individuals were identified as regular users. The mean age of those affected was 18.3 years. Findings indicate that children are particularly vulnerable to accidental poisoning from psychoactive substances, predominantly due to the ingestion of delta-9-tetrahydrocannabinol (THC), the principal psychoactive compound. The clinical manifestations vary depending on the dosage and pattern of use. Out of 438 cases with documented outcomes, six resulted in fatalities, while the remainder showed improvement following appropriate medical interventions, including gastrointestinal decontamination.

**Conclusions:**

The escalating use of cannabis necessitates immediate and strategic public health interventions, particularly targeting the youth, to mitigate its adverse health effects and societal impact. It is crucial to develop targeted strategies to enhance preventive efforts and curb cannabis misuse among vulnerable populations.

**Disclosure of Interest:**

None Declared

